# Supplementary material for: Effects of Hydrogen Sulfide on Sugar, Organic Acid, Carotenoid, and Polyphenol Level in Tomato Fruit
Source: Plants (Basel). 2023 Feb 6;12(4):719. doi: 10.3390/plants12040719 (PMC9965552; doi:10.3390/plants12040719)
Supplement: Supplementary file 1 [file plants-12-00719-s001.zip › Supplementary Table S1.pdf]

plants

MDPI

Supplementary Table S1. Primers used for RT-qPCR assays.

Gene name	Accession NO.	Forward primer (5′-3′)	Reverse primer (5′-3′)
PFPP	101243849	GAGATCCACACGGCAATGTCCAG	CCTTCTGCTTCCTCTGATCCAACTC
ATPPF	101245480	CACGGCTTCTTGCTTCAACCAATC	CTTTGTGAGTGCTGTCGCTTATGC
FBA6	101243907	TAACATGGTCACCCCTGGCTCTG	CCTCTTCCTCACTCTGTCCTCCTG
GPI	101262594	CATTCGCTTTCTGGGACTGGGTAG	ATGGTGCTGAGAGGAAATGCTGATC
GPD1	101249507	GCCGTATTCGTGGGTATCTTTCTCG	TTCGTGCTCAGATATTGCCTTGTCC
POL2	101259276	TGCTCTCACCCTCATCGGACAAG	CCTCCTCGGAATCGTACAGTTTCAC
MDH	543805	ATGAGGTGTTCCAGGATGCAGAATG	TCCAATAAGCCAGCTCGTTCCATAC
ME1	544164	TCCCTCCTCTCATCCTCCCTCTC	CCCAGCTCCAGAAGATCCAATCAAG
ME2	543522	GCTCTCTCAAACCCAACCTCACAAG	CTCATTGACACTGCGTACCTCACTC
CSG	101260902	ACAGCATTCAGCAGTTCCACAAGG	AAGCATTAGCGGGTGACGAATCTG
CS3	101258079	TGCGATGGAGAACAAGTTGACTCTG	TGCTTGGTGCCTGCCTATTATTGG
CSM	101249011	GTCTTTCTCCGCCTCTGTCTGTTTG	GCTCGGCAATATGGGATCGTGAC
PAL5	101244220	GCCATCTAATCTCACAGCAGGAAGG	AAGTTCCGAGCAGTAAGAAGCCATC
4CL	101249624	ACCGCCTATTGTTCTCGCCATTG	GCTCGGACGGCATCTTCAAGTTC
MDHAR	778288	TGCTTCTCCACCATAACCCATTGC	GCGCGAAAGTTCAACAAGATCAGTG
DHAR1	778229	AAGAAGTGGAGTGTGCCTGAAAGC	CACGCATACAAGGACACGGTGAG
ZDS	543629	CCAGTGGTGCGGGCTTTAGTTG	CAAGTAGTGCGGAGAGGACAGAAAC
CCD7	100313501	CCATACCATCCGATTTCCCTTCCG	TCCTTAAGTAGCCGTGTCCGTCTAG
CrtL-e-1	544129	GTCCTGCTGGTCTTGCTCTTGC	CTTTGAACTCGTCCTCCCATACACC
psy1	543988	TTATGTTGCTGGTACGGTTGGGTTG	CGCTCTCTGTTGTTGCCTTTGATTC
LCY1	544104	GTTGTTGTGGATCTTGCTGTGGTTG	AGAGAGTCCTGCTTCAGAAACTTGC
PDS	544073	TTATCTCGCAAGTGTGGCTATGGTG	GAAGATGCCTGCTTTCGGTGATTTG
CHS1	778294	ATGGTCACCGTGGAGGAGTATCG	AGGCGTAGATGTTCCAATGGCTAAG
CHS2	778295	GCCGACTACCAACTCACCAAGC	TCCCACCAGCAAAGCAACCTTG
F3H	100736482	GAGGCAATGGGCTTAGAGAAAGAGG	GGTAATGGTTCCTGGATCGGTGTG
FLS	102577717	ACCTTGAATGAGTCGGTGCTTGTG	GAGCCAGATTGTGGTCAGGTGTTC
ACTIN	NC_015447.3	AATGAACTTCGTGTGGCTCCAGAG	ATGGCAGGGGTGTTGAAGGTTTC
